# Clinical and Genetic Features of Tubulointerstitial Nephritis and Uveitis Syndrome with Long-Term Follow-Up

**DOI:** 10.1155/2018/4586532

**Published:** 2018-04-16

**Authors:** Hiroaki Kanno, Kyoko Ishida, Wataru Yamada, Ikumi Shiraki, Hiroki Murase, Yuka Yamagishi, Kiyofumi Mochizuki

**Affiliations:** ^1^Department of Ophthalmology, Gifu University Graduate School of Medicine, 1-1 Yanagido, Gifu-shi, Gifu 500-1194, Japan; ^2^Department of Ophthalmology, Toho University Ohashi Medical Center, 2-17-6, Ohashi, Meguro-ku, Tokyo 153-8515, Japan; ^3^Murase Eye Clinic, 17-1 Hanaike, Hagiwaracho, Gero-shi, Gifu 509-2515, Japan; ^4^Department of Infection Control and Prevention, Aichi Medical University, 1-1 Karimata, Iwasaku, Nagakute-shi, Aichi 480-1195, Japan

## Abstract

**Purpose:**

To investigate the clinical manifestations, prognosis, and HLA-type of tubulointerstitial nephritis and uveitis syndrome (TINU) with long-term follow-up.

**Methods:**

Clinical data of five patients with TINU were retrospectively reviewed.

**Results:**

The mean age was 15.8 years. The mean follow-up periods were 54.0 months. The initial subjective symptoms were bulbar injection (100%), ocular pain (80%), and blurred vision (60%). The medical department that the patients visited first was ophthalmology in 4 (80%) cases. Urinalysis showed the characteristic increase of the *β*2 microglobulin in all (100%) patients. Uveitis and nephritis were diagnosed within 1 week from each other. Although two showed recurrences, the topical and systemic steroid treatment with mean duration of 14.1 months brought the resolution of nephritis and uveitis in all patients. Recurrence-free periods ranged from 12 to 71 months. The final visual outcome was 20/20 or better in all cases. HLA-DR4 or the allele of DRB1^∗^04 was present in all (100%) patients.

**Conclusions:**

TINU should be considered in the differential diagnosis in young patients with uveitis of unknown origin and renal dysfunction. Urinary *β*2 microglobulin level and HLA typing may help in the diagnosis of TINU. The prognosis for patients with TINU is generally good with steroid treatment.

## 1. Introduction

The tubulointerstitial nephritis and uveitis syndrome (TINU) is a specific form of intraocular inflammation (uveitis) combined with kidney disease that was first described in 1975 by Dobrin et al. [1] in two patients. It affects approximately 1-2% of the patients who visit uveitis clinics and, however, may represent up to a third of bilateral acute anterior uveitis in patients younger than 20 years old [2–6]. In a prospective multicenter epidemiological study of uveitis in Japan in 2009, TINU accounted only for 0.4% of the 2556 patients with uveitis [7]. Since TINU is considered relatively to be a rare disease, it is still unfamiliar to most ophthalmologists, pediatricians, or nephrologists [2, 4].

Generally, renal dysfunction in TINU is mild, and uveitis tends to recur [8, 9]. Patients younger than 20 years of age are more prone to chronic uveitis than the adult population [8]. Although the uveitis can relapse during and after therapy, long-term follow-up reports are few and lacking (The mean follow-up of 17.8 [10], 19.6 [11], 25.4 [5], and 54.8 months [12]).

The pathogenesis of TINU is still unknown. TINU has been reported to have an association with human leukocyte antigen (HLA), and HLA alleles have been implicated as susceptibility risk factors [8, 13–16]. However, the distribution of the HLA haplotypes varies considerably in Japan [3, 17].

We had 5 cases of TINU with a mean age of 15.8 years old and a mean follow-up of 54.0 months. TINU is a rare disease [4]; however, it may be far more common than currently appreciated, especially in young patients whom mild renal disease does not become symptomatic and/or if diagnostic tests regarding renal involvement are not performed at the time of presentation. Moreover, no guidelines or recommendations for screening have been made to date in patients with interstitial nephritis [2]. In this report, we described clinical findings, long-term treatment outcomes, and the HLA haplotype in TINU cases.

## 2. Materials and Methods

This was a retrospective study of 5 patients with TINU who were examined in the Department of Ophthalmology of Gifu University Graduate School of Medicine or branch hospital, the Chuno Kosei Hospital between January 2001 and December 2015. The study followed the tenets of the Declaration of Helsinki and was approved by the affiliated hospitals' institutional review boards.

The diagnostic criteria for TINU were based on the reports of Mandeville et al. [8] and Mackensen and Billing [4]. Two patients with definite TINU and 3 with probable TINU were included in the study. Patients with sarcoidosis, Behçet's disease, Vogt-Koyanagi-Harada disease, Wegener's granulomatosis, and juvenile idiopathic arthritis-associated uveitis were excluded [2]. The following information was collected from their medical records with special attention given to the gender, age, initial manifestations, ocular findings, laboratory findings, treatment, visual prognosis, follow-up and treatment periods, and HLA typing.

## 3. Results

### 3.1. Basic Demographics (Table 1)

There were 2 males (40%) and 3 females (60%). Patient age at the first visit to the hospital ranged from 13 years to 24 years (mean 15.8 years, median 14 years). The ages of four patients (80%) were between 13 and 15 years. One of the patients had diabetes, and the others did not have any systemic diseases.

### 3.2. Initial Signs and Symptoms (Table 1)

The clinical department that the patients first visited for detailed examinations was ophthalmology in 4 (80%) and pediatrics in 1 (20%) patient. The median interval between the onset of subjective symptoms and the day that patients first visited the hospital was 7 days (range 7–90 days). All patients reported sudden onset of disease and initially had subjective ocular symptoms. The initial signs and symptoms were eye redness in 5 (100%), eye pain in 4 (80%), and a decrease in vision in 3 patients (60%). Both eyes were affected in all patients (100%). None had general symptoms, for example, fatigue, weight loss, fever, anorexia, abdominal or flank pain, and arthralgias at the onset of this disease [4]. However, two patients (cases 1 and 3) had been incidentally found to have proteinuria without any general symptoms by annual medical examinations at school or work, before the appearance of subjective eye symptoms.

### 3.3. Ocular Findings (Table 1)

The initial visual acuity was better than 20/20 in 7 eyes (70%) and deteriorated to 12/20 in one eye (10%) of the patients. The initial intraocular pressure (IOP) was lower than 15 mmHg in all eyes. Anterior segment findings included conjunctival injection, iridocyclitis, and fine keratic precipitates in all patients (100%), but severe inflammation with more than 3+ cells (by the grading of inflammation described by the SUN working groups) [18] was not seen. Posterior synechiae were seen in one patient (20%, case 3). Posterior segment findings included cells in the anterior vitreous (40%, cases 3 and 4), peripheral vasculitis (20%, case 4), retinal exudates (40%, cases 3 and 4), and optic disk swelling (40%, cases 3 and 4).

### 3.4. Laboratory Findings (Table 1)

Blood tests and urinalysis were administered to investigate the cause of uveitis. The blood counts and liver function were normal. The blood urea nitrogen (BUN) level was normal in all patients, and the serum creatinine level was slightly increased at 1.0 mg/dl (normal range 0.3 to 0.9 mg/dl) in 2 (40%) patients. Antinuclear antibody and rheumatoid factor were negative in all patients. The level of angiotensin-converting enzyme was normal, and hepatitis B and C antibodies were not detected. The estimated glomerular filtration rate (eGFR) was outside normal limits in 2 (40%) patients (normal range 60 ≤ eGFR < 90 mL/mim/1.73m^2^).

Urinalysis was positive for proteinuria in 3 patients (60%) and positive for glycosuria in 4 patients (80%). The urinal *β*2 microglobulin was increased in all patients (100%), with values at least 10 times higher than upper normal limits (<230 *μ*g/L). N-Acetylglucosaminidase (normal range <7.0 U/L) was increased in 3 patients (75%, not measured in one patient). Chest X-rays and computed tomography scans were normal in all cases.

### 3.5. Renal Biopsy, Classification, and Cause (Tables 1 and 2)

All patients have consulted pediatricians or nephrologists after abnormal urinary test. Two patients (cases 1 and 3) had a renal biopsy, and the histopathological findings showed focal tubulointerstitial inflammatory infiltrations containing lymphocytes and nonspecific histiocytes. From these results, these 2 patients (cases 1 and 3) were confirmed to have definite TINU. The 3 other patients (cases 2, 4, and 5) were diagnosed with probable TINU. They did not receive a renal biopsy because pediatricians did not justify this invasive procedure for renal dysfunction level in each patient. The uveitis and nephritis were diagnosed within 1 week from each other in all patients.

TINU may be triggered by infectious disease and numerous medications, but the causes were unknown in all cases.

### 3.6. Treatment and Outcomes (Table 2)

Depending on the severity of the disease conditions, topical corticosteroids were used to treat the anterior uveitis and systemic steroids were used for renal indications and/or the posterior uveitis. Two (40%, cases 2 and 5) of 5 patients without posterior uveitis were treated with topical or subconjunctival steroids alone, and the remaining 3 (60%, cases 1, 3, and 4) patients had the topical steroids combined with systemic corticosteroids prescribed by internists or pediatricians mainly for the tubulointerstitial nephritis. Initial dose of corticosteroid determined by internists or pediatrician ranged from 15 to 60 mg (15 mg for case 1, 30 mg for case 3, and 60 mg for case 4).

After a reduction of the steroid dosage, two patients (40%, cases 3 and 4) who had developed complicated posterior segment findings had a recurrence or exacerbation of the nephritis. Numbers of recurrence or exacerbation were none in 3 patients (cases 1, 2, and 5), twice in case 3, and once in case 4, respectively. Dose of systemic steroids was increased whenever a recurrence or exacerbation was observed. One patient (case 4) developed ocular hypertension during the topical steroid therapy. Average follow-up periods were 54.0 months (range 23–86 months). The mean number of attack per year was 1.6 in the 1st year and 0 thereafter. After the mean steroid treatment duration of 14.1 months (range 11–18 months), no recurrences were observed. The nephritis and uveitis were resolved in all patients, and the last recorded visual acuity was at least 20/20 in both eyes in all patients. Recurrence-free periods ranged from 12 to 71 months.

### 3.7. HLA Types (Table 2)

The A locus had A24, 26, and 31 in 2 (40%) patients, and the DR locus had DR4 in 1 (20%) patient. The DRBI allele was DRB1^∗^01 in 2 (40%) patients and DRB1^∗^04 in 4 (80%) patients. The DQ was not tested in 3 patients and was DQ A1^∗^01/B1^∗^05 in 1 patient.

## 4. Discussion

Mandeville et al. [8] were the first to provide structured diagnostic criteria for TINU. Definite TINU syndrome is diagnosed when acute tubulointerstitial nephritis (TIN) is firmly established and the patient has bilateral anterior uveitis of sudden onset. Acute TIN is diagnosed either by histologic examination of renal biopsy specimens or by all three criteria as follows: (1) abnormal renal function (increased serum creatinine or decreased creatinine clearance); (2) abnormal urinalysis: increased *β*2 microglobrin, low-grade proteinuria, presence of urinary eosinophils, pyuria or hematuria without infection, urinary white cell casts, or normoglycemic glucosuria; and (3) asystemic illness lasting ≥ 2 weeks, characterized by a combination of the following symptoms and laboratory findings: (a) signs and symptoms: fever, weight loss, anorexia, malaise, fatigue, rash, abdominal or flank pain, arthralgias, or myalgias; and (b) laboratory findings such as the evidence of anemia, abnormal liver function, eosinophilia, or a Westegren erythrocyte sedimentation rate > 40 mm/h. When two of the 3 criteria are fulfilled and typical uveitis is present, a diagnosis is probable for TINU. When one of the clinical findings meets the criteria and typical uveitis coexists, a diagnosis is possible for TINU [4]. We diagnosed TINU in our five patients using their criteria [4, 8]. Since the renal function does not change significantly between patients with and without treatment with prednisolone, a renal biopsy as an invasive diagnostic method remains controversial [12, 19]. According to Goda et al.'s data [3], all (100%) of the 12 patients with biopsy-proven TINU met criterion 2 for abnormal urinalysis (all patients had increased urinary *β*2 microglobulin), but only 3 (25%) met criterion 1 (increased serum creatinine). Similar to them, all (100%) patients had increased urinary *β*2 microglobulin, but 2 (40%) patients had increased serum creatinine in our cases. Mackensen et al. [20] reported that creatinine levels were more likely to be elevated in patients older than 40. Average age of patients in Goda et al.'s [3] and our study were 21.2 and 15.8, respectively. The sensitivity and specificity of elevated *β*2 microglobulin were 87.5% and 70%, respectively, with positive predictive values and negative predictive values of urinary *β*2 microglobulin greater than 1000 *μ*g/L being 88 and 97%, respectively [21]. Takemura et al. [22] reported that the histologic grade of TIN was correlated with urinary *β*2 microglobulin levels. Urinary *β*2 microglobulin is a very sensitive marker for tubular damage, and its analysis is helpful in the diagnosis of TINU syndrome [4, 20, 21], especially when a renal biopsy is not indicated.

We had only 5 cases in 15 years at our medical institutions that provide medical support to a population of approximately 500,000. The median age of the onset of TINU is 15 years and is dominant in young girls in the Mandeville et al.'s review [8]. In our cohort, the median age was 14 years, and 3 (60%) of the 5 were girls. Ocular effects seen with uveitis are limited to the anterior chamber in 80% of cases; both eyes are affected in 77% of cases in Aguilar et al.'s review [6]. In our cases, anterior uveitis and bilateral uveitis were observed in 60% and 100%, respectively.Uveitis in TINU syndrome may cause severe complications in approximately 20% of patients, the most common being posterior synechia in about 70% of patients, optic disc swelling in 25%, cataract in 20%, and secondary glaucoma in 20% [8]. Complications including posterior synechiae, optic disk swelling, and secondary glaucoma were observed in two (40%) of five patients in our cohorts.

Uveitis may occur before the presentation of acute interstitial nephritis in approximately 20% of cases, but in most cases (65%), nephritis precedes uveitis, and in 15%, the two conditions occur simultaneously [8]. In our cohort, two (40%) patients were incidentally pointed to have proteinuria by annual medical examinations at school or work. However, eye redness was the most common subjective symptom before abnormal results of urea were seen upon precise testing at the hospital. Asystemic illness lasting ≥ 2 weeks was only observed in 33% in Goda et al.'s biopsy-proven Japanese TINU cases [3], and none in our cohorts. On the other hand, nineteen Finnish children with a biopsy-proven TIN had no ocular symptom in 50%, fatigue in 95%, and loss of appetite in 84% of patients [5]. Thus, most of the Japanese patients might initially present with ophthalmic findings or mild symptoms of TIN of which they are unaware. In fact, 4 of 5 patients visited an ophthalmologist first in our cohort. Thus, ophthalmologists play an important role in the initial diagnosis of patients with the TINU syndrome. TINU should be considered in the differential diagnosis especially in young patients with anterior uveitis of unknown origin.

In general, TINU responds to steroids. Topical corticosteroids were used to treat the anterior uveitis, and systemic steroids were used for renal indications and/or the posterior uveitis [2]. TIN can be resolved spontaneously and dialysis therapy is not usually required [23]. Steroid therapy (1 mg/kg per day for 2-3 weeks with subsequent taper) is often administered when renal function impairment is severed or prolonged [22]. Few cases characterized by prolonged or recurrence of interstitial nephritis are treated with high-dose corticosteroids or steroid-sparing immunosuppressives [24]. Legendre et al. [10] reported that the median number of attack per patient per year was 1.0 (range 0.1–4.6) for eye symptoms and 0.8 (range 0.2–2.0) for renal symptoms in cases with French adult TINU. According to the review of 133 cases with TINU, 54% of patients developed recurrences, 17% were treated with topical steroids alone, 80% required systemic steroids, and 9% needed immunosuppressive treatment [8]. In our cases, recurrences or exacerbation were found in two (40%) cases; however, all cases were resolved with systemic steroids by the end of the observation periods. Since the inflammation in TINU syndrome can relapse during and after therapy, a long-term follow-up is required. The prolonged use of adequate treatment with the mean treatment duration of 29.5 months (range 13–40 months) improved the recurrence-free periods [12]. On the other hand, patients were needed on a low-dose topical treatment regime for months but this was hardly ever necessary for more than a year [4]. After the mean treatment duration of 14.1 months (range 11–18 months) in our cases, TINU had not relapsed for more than 12 months (range 12 to 71 months). Further investigations with longer follow-up periods are needed to clarify the optimum treatment regime.

The pathogenesis of TINU is not clear; however, it is thought to be the result of an autoimmune process that might involve cellular and humoral autoimmunity [13, 25]. Autoimmune diseases such as rheumatoid arthritis, thyroidism, and Sjögren syndrome, infectious and iatrogenic factors have been reported to accompany TINU cases [2, 8, 20, 21]. We did not find such association in our case.

Several studies have reported that renal tubulointerstitial infiltrations are composed of activated lymphocytes, predominantly helper/inducer T-cell subset [26, 27]. Immunohistochemical kidney analysis in TINU patients demonstrated that the interstitium is infiltrated mainly by T-cells, together with monocytes/macrophages [22, 28]. Otherwise, ciliary body and renal tubular epithelium share some similar functions including those pertaining to electrolyte transporters sensitive to carbonic anhydrase inhibitors [8]. Thus, they might share cross-active autoantigens. In fact, the high prevalence of serum anti-modified CRP (mCRP) autoantibodies were detected in TINU syndrome in active phase of nephritis [25]. Tan et al. [25] also found that the staining of mCRP and human IgG was colocalized in renal and ocular tissues. Although, these findings are impressive, we have no data of mCRP in our patients.

Genetic markers for TINU also have been reported to be related to the risk of development of the syndrome. Loss of T-cell tolerance is suggested by several reports identifying a strong link between TINU and human leukocyte antigen (HLA) [15, 16]. In the report of Goda et al. [3], 7 (87.5%) of 8 patients in Hokkaido, Japan, were HLA-A2, 3 (37.5%) of 8 patients were HLA-A24, 2 (25%) of 8 patients were HLA-A31, and 2 (25%) of 8 patients were HLA-DR4. Mandeville et al. [8] reported that HLA-A2 and HLA-A24 were important antigens associated with TINU in Japanese individuals. On the other hand, Matsumoto et al. [17] compared 22 Japanese adult cases of previous reported TINU to 50 healthy Japanese adults and concluded that there were no significant differences in HLA-A2 and HLA-A24 between those with and without the disease. In our cases, the A locus was A24, 26, and 31 in 2 (40%) patients, and A2 in 1 (20%).

Levinson et al. [15] found that HLA-DQA1^∗^01, HLA-DQB1^∗^05, and HLA-DQB1^∗^01 were associated with TINU and concluded that HLA-DQA1^∗^01/HDQB1^∗^05 may be related to the risk of development of the disease in their population. We found DQ A1^∗^01/B1^∗^05 in one patient with the definite type.

The HLA-DR4 serotype, and its corresponding allele HLA-DRB1^∗^04, has been reported to be a risk factor for Vogt-Koyanagi-Harada (VKH) disease [29]. In our cohorts, HLA-DR4 or the allele of DRB1^∗^04 was present in all (100%) patients. It was also reported that VKH patients are sensitized to melanocyte epitopes [30]. HLA-DR is an MHC class II cell surface receptor which displays peptide antigens produced from the HLA-DRB1 gene to the immune system. If the immune system recognizes a peptide induced by a virus, bacteria, or environmental substances, it triggers a response to attack these invading factors [24]. A common antibody may be produced against ocular and renal proteins, subsequently a crossed autoimmune reaction could induce the renal and ocular alterations in TINU. HLA-DR4 or the allele of DRB1^∗^04 may be involved in these reactions. HLA typing may be helpful in diagnosing TINU; however, further research on this association is required.

Our study has several limitations. First, its retrospective nature may have introduced a degree of bias. Second, as mentioned earlier, it might have been underdiagnosed if mild renal disease does not become symptomatic and/or if diagnostic tests regarding renal involvement are not performed at the time of presentation. Prospective long-term follow-up of more subjects is needed as a creation of the guideline for screening and treatment strategy in patients with TINU.

In conclusion, we reported clinical findings, treatment outcomes, and the HLA haplotype in 5 cases of TINU syndrome with a mean follow-up of 54.0 months. Although recurrences or exacerbation is observed, the prognosis for patients with TINU is good with steroid treatment. Since some Japanese patients might initially present with ophthalmic findings or mild symptoms of TIN of which they are unaware, TINU should be considered in the differential diagnosis especially in young patients with anterior uveitis of unknown origin and renal dysfunction. Examination for urinary *β*2 microglobulin and HLA typing may be helpful in diagnosing TINU.

## Figures and Tables

**Table 1:** Patient data 1.

Case	Type	Age	Gender	Side	Family history	Days between the first sign of uveitis and consultation (day)	Presenting symptoms	First sign uveitis or TIN	Uveitis		Visual acuity			IOP	Urinalysis		Blood test		Renal biopsy
									Anterior	Posterior	Initial		Last	Initial (mmHg)	Initial *β*2-MG (*μ*g/L)	Initial NAG (U/L)	Initial Cr (mg/dl)	Initial eGFR (l/min/1.73m^2^)	
1	Definite	24	Female	Both	Grandmother: renal failure Brother: diabetes mellitus	9	Eye pain Eye redness	TIN (proteinuria)	+	−	R L	30/20 30/20	30/20 30/20	10 10	32700	20.6	1.00	57.6	+
2	Probable	14	Female	Both	Mother: glaucoma	7	Eye pain eye Redness decreased in vision	Uveitis	+	−	R L	14/20 30/20	30/20 30/20	9 9	2340	13.8	0.65	105.6	−
3	Definite	13	Female	Both	−	90	Eye redness Decreased in vision	TIN (proteinuria)	+ Posterior synechiae	Cells in the anterior vitreous, retinal exudates, and optic disk swelling	R L	18/20 12/20	30/20 24/20	11 11	2630	5.2	0.80	87.7	+
4	Probable	15	Male	Both	−	7	Eye pain Eye redness decreased in vision	Uveitis	+	Cells in the anterior vitreous, vasculitis, and optic disk swelling	R L	20/20 20/20	30/20 24/20	10 11	9600	19.5	1.00	89.2	−
5	Probable	13	Male	Both	−	2	Eye pain Eye redness	Uveitis	+	−	R L	30/20 30/20	30/20 30/20	13 11	3460	n.d.	0.80	n.d.	−

TIN: tubulointerstitial nephritis; IOP: intraocular pressure; R: right eye; L: left eye; n.d.: not done; *β*2-MG: *β*2 microglobulin; NAG: N-acetylglucosaminidase; Cr: creatinine; eGFR: estimated glomerular filtration rate.

**Table 2:** Patient data 2.

Case	Type	Steroid			Secondary glaucoma	Recurrence	Last follow-up data		Follow-up (months)	HLA				
		Topical	Subconjunctival	Systemic (initial dose) [dose after recurrence]			Blood test Cr (mg/dl)	Urinalysis *β*2-MG (*μ*g/L)		HLA-A	HLA-B	HLA-C	HLA-DQ	HLA-DR
1	Definite	+	−	+ (15 mg)	−	−	0.69	n.d.	23	A24:02 A26:01 : 01	B40:02 : 01 B48:01 : 01	C03:04 : 01 C08:03 : 01	DQA1^∗^03 : 01 : 01 DQA1^∗^03 : 03 DQB1^∗^03 : 02 : 01 DQB1^∗^04 : 01 : 01	DRB1^∗^04 : 05 DRB1^∗^08 : 02 DPB1^∗^02 : 02 DPB1^∗^05 : 01
2	Probable	+	+	−	−	−	0.49	54	47	A31	B60/B55	−	−	DRB1^∗^04 DRB1^∗^14
3	Definite	+	−	+ (30 mg) [80 mg] [80 mg]	−	3 and 6 months	0.50	125	86	A2/A31	B7/B40	−	DQA1^∗^01 DQA1^∗^03 DQB1^∗^05 DQB1^∗^04	DRB1^∗^01 DRB1^∗^04
4	Probable	+	+	+ (60 mg) [20 mg]	+	3 months	0.70	94	69	A11	B54(22)/B35	Cw1/Cw3	−	DR4
5	Probable	+	−	−	−	−	0.70	102	46	A24/A26	B7/B56	C0401 C0702	−	DRB1^∗^01 DRB1^∗^04

n.d.: not done; *β*2-MG: *β*2 microglobulin.

## References

[1] Dobrin R. S., Vernier R. L., Fish A. J. (1975). Acute eosinophilic interstitial nephritis and renal failure with bone marrow-lymph node granulomas and anterior uveitis. *The American Journal of Medicine*.

[2] Pakzad-Vaezi K., Pepple K. L. (2017). Tubulointerstitial nephritis and uveitis. *Current Opinion in Ophthalmology*.

[3] Goda C., Kotake S., Ichiishi A., Namba K., Kitaichi N., Ohno S. (2005). Clinical features in tubulointerstitial nephritis and uveitis (TINU) syndrome. *American Journal of Ophthalmology*.

[4] Mackensen F., Billing H. (2009). Tubulointerstitial nephritis and uveitis syndrome. *Current Opinion in Ophthalmology*.

[5] Saarela V., Nuutinen M., Ala-Houhala M., Arikoski P., Rönnholm K., Jahnukainen T. (2013). Tubulointerstitial nephritis and uveitis syndrome in children: a prospective multicenter study. *Ophthalmology*.

[6] Aguilar M. C., Lonngi M., de-la-Torre A. (2016). Tubulointerstitial nephritis and uveitis syndrome: case report and review of the literature. *Ocular Immunology and Inflammation*.

[7] Ohguro N., Sonoda K. H., Takeuchi M., Matsumura M., Mochizuki M. (2012). The 2009 prospective multi-center epidemiologic survey of uveitis in Japan. *Japanese Journal of Ophthalmology*.

[8] Mandeville J. T. H., Levinson R. D., Holland G. N. (2001). The tubulointerstitial nephritis and uveitis syndrome. *Survey of Ophthalmology*.

[9] Han J. M., Lee Y. J., Woo S. J. (2012). A case of tubulointerstitial nephritis and uveitis syndrome in an elderly patient. *Korean Journal of Ophthalmology*.

[10] Legendre M., Devilliers H., Perard L. (2016). Clinicopathologic characteristics, treatment, and outcomes of tubulointerstitial nephritis and uveitis syndrome in adults: a national retrospective strobe-compliant study. *Medicine*.

[11] Gion N., Stavrou P., Foster C. S. (2000). Immunomodulatory therapy for chronic tubulointerstitial nephritis–associated uveitis. *American Journal of Ophthalmology*.

[12] Sobolewska B., Bayyoud T., Deuter C., Doycheva D., Zierhut M. (2016). Long-term follow-up of patients with tubulointerstitial nephritis and uveitis (TINU) syndrome. *Ocular Immunology and Inflammation*.

[13] Gafter U., Kalechman Y., Zevin D. (1993). Tubulointerstitial nephritis and uveitis: association with suppressed cellular immunity. *Nephrology Dialysis Transplantation*.

[14] Gorroño-Echebarría M. B., Calvo-Arrabal M. A., Albarrán F., Alvarez-Mon M. (2001). The tuberculointerstitial nephritis and uveitis (TINU) syndrome is associated with HLA-DR14 in Spanish patients. *The British Journal of Ophthalmology*.

[15] Levinson R. D., Park M. S., Rikkers S. M. (2003). Strong associations between specific HLA-DQ and HLA-DR alleles and the tubulointerstitial nephritis and uveitis syndrome. *Investigative Ophthalmology & Visual Science*.

[16] Mackensen F., David F., Schwenger V. (2011). HLA-DRB1^∗^0102 is associated with TINU syndrome and bilateral, sudden-onset anterior uveitis but not with interstitial nephritis alone. *The British Journal of Ophthalmology*.

[17] Matsumoto K., Fukunari K., Ikeda Y. (2015). A report of an adult case of tubulointerstitial nephritis and uveitis (TINU) syndrome, with a review of 102 Japanese cases. *American Journal of Case Reports*.

[18] Jabs D. A., Nussenblatt R. B., Rosenbaum J. T., Standardization of Uveitis Nomenclature (SUN) Working Group (2005). Standardization of uveitis nomenclature for reporting clinical data. results of the first international workshop. *American Journal of Ophthalmology*.

[19] Jahnukainen T., Rönnholm K., Ala-Houhala M., Nuutinen M. (2014). Corticosteroid therapy can be delayed but not omitted in idiopathic tubulointerstitial nephritis. *Pediatric Nephrology*.

[20] Mackensen F., Smith J. R., Rosenbaum J. T. (2007). Enhanced recognition, treatment, and prognosis of tubulointerstitial nephritis and uveitis syndrome. *Ophthalmology*.

[21] Hettinga Y. M., Scheerlinck L. M. E., Lilien M. R., Rothova A., de Boer J. H. (2015). The value of measuring urinary *β*2-microglobulin and serum creatinine for detecting tubulointerstitial nephritis and uveitis syndrome in young patients with uveitis. *JAMA Ophthalmology*.

[22] Takemura T., Okada M., Hino S. (1999). Course and outcome of tubulointerstitial nephritis and uveitis syndrome. *American Journal of Kidney Diseases*.

[23] Savaj S., Asgari M. (2011). Tubulointerstitial nephritis and uveitis: report of a rare syndrome. *Iranian Journal of Kidney Diseases*.

[24] Hinkle D. M., Foster C. S. (2008). Tubulointerstitial nephritis and uveitis syndrome. *International Ophthalmology Clinics*.

[25] Tan Y., Yu F., Qu Z. (2011). Modified C–reactive protein might be a target autoantigen of TINU syndrome. *Clinical Journal of the American Society of Nephrology*.

[26] Tanaka H., Suzuki K., Nakahata T., Tateyama T., Waga S., Ito E. (2001). Repeat renal biopsy in a girl with tubulointerstitial nephritis and uveitis syndrome. *Pediatric Nephrology*.

[27] Abed L., Merouani A., Haddad E., Benoit G., Oligny L. L., Sartelet H. (2007). Presence of autoantibodies against tubular and uveal cells in a patient with tubulointerstitial nephritis and uveitis (TINU) syndrome. *Nephrology Dialysis Transplantation*.

[28] Kobayashi Y., Honda M., Yoshikawa N., Ito H. (1998). Immunohistological study in sixteen children with acute tubulointerstitial nephritis. *Clinical Nephrology*.

[29] Shindo Y., Ohno S., Yamamoto T., Nakamura S., Inoko H. (1994). Complete association of the HLA-DRB1^∗^04 and -DQB1^∗^04 alleles with Vogt-Koyanagi-Harada’s disease. *Human Immunology*.

[30] Damico F. M., Cunha-Neto E., Goldberg A. C. (2005). T-cell recognition and cytokine profile induced by melanocyte epitopes in patients with HLA-DRB1^∗^0405-positive and -negative Vogt-Koyanagi-Harada uveitis. *Investigative Ophthalmology & Visual Science*.

